# Integrin-linked kinase-frizzled 7 interaction maintains cancer stem cells to drive platinum resistance in ovarian cancer

**DOI:** 10.21203/rs.3.rs-4086737/v1

**Published:** 2024-03-13

**Authors:** Rula Atwani, Amber Rogers, Rohit Nagare, Mayuri Prasad, Virginie Lazar, George Sandusky, Fabrizio Pin, Salvatore Condello

**Affiliations:** Indiana University School of Medicine

**Keywords:** beta-catenin, cancer stem cells, frizzled 7, integrin-linked kinase, ovarian cancer, chemoresistance, tumor microenvironment

## Abstract

**Background:**

Platinum-based chemotherapy regimens are a mainstay in the management of ovarian cancer (OC), but emergence of chemoresistance poses a significant clinical challenge. The persistence of ovarian cancer stem cells (OCSCs) at the end of primary treatment contributes to disease recurrence. Here, we hypothesized that the extracellular matrix protects CSCs during chemotherapy and supports their tumorigenic functions by activating integrin-linked kinase (ILK), a key enzyme in drug resistance.

**Methods:**

TCGA datasets and OC models were investigated using an integrated proteomic and gene expression analysis and examined ILK for correlations with chemoresistance pathways and clinical outcomes. Canonical Wnt pathway components, pro-survival signaling, and stemness were examined using OC models. To investigate the role of ILK in the OCSC-phenotype, a novel pharmacological inhibitor of ILK in combination with carboplatin was utilized in vitro and in vivo OC models.

**Results:**

In response to increased fibronectin (FN) secretion and integrin β1 clustering, aberrant ILK activation supported the OCSC phenotype, contributing to OC spheroid proliferation and reduced response to platinum treatment. Complexes formed by ILK with the Wnt receptor frizzled 7 (Fzd7) were detected in tumors and showed a strong correlation with metastatic progression. Moreover, TCGA datasets confirmed that combined expression of ILK and Fzd7 in high grade serous ovarian tumors is correlated with reduced response to chemotherapy and poor patient outcomes. Mechanistically, interaction of ILK with Fzd7 increased the response to Wnt ligands, thereby amplifying the stemness-associated Wnt/β-catenin signaling. Notably, preclinical studies showed that the novel ILK inhibitor compound 22 (cpd-22) alone disrupted ILK interaction with Fzd7 and CSC proliferation as spheroids. Furthermore, when combined with carboplatin, this disruption led to sustained AKT inhibition, apoptotic damage in OCSCs and reduced tumorigenicity in mice.

**Conclusions:**

This “outside-in” signaling mechanism is potentially actionable, and combined targeting of ILK-Fzd7 may represent a new therapeutic strategy to eradicate OCSCs and improve patient outcomes.

## Background

Ovarian cancer (OC) is the fifth leading cancer among women in the US (1). Intra-peritoneal (ip) metastasis and chemoresistance represent major causes of poor clinical outcome (2). Both recurrence and spread have been linked to ovarian cancer stem cells (OCSCs), which are resistant to traditional chemotherapy and possess the capacity to undergo either symmetric or asymmetric division, recapitulating hierarchically organized tumor phenotype when injected in NOD/SCID/IL2rγnull (NSG) mice (2, 3). Successful identification of OCSCs involves the utilization of CD133 (prominin-1) (4), CD44 (hyaluronic acid receptor)/CD117 (c-Kit) (5) and increased metabolic activity of aldehyde dehydrogenase (ALDH) (4, 6). The ALDH1A1 isoform in particular has been widely used to identify and characterize CSCs in OC and other solid tumors (7). However, effective treatment strategies targeting OCSCs remain to be developed.

Advanced OC is commonly associated with the accumulation of peritoneal ascitic fluid (8). Ascites is enriched in cytokines (9), growth factors (10), and extracellular macromolecules such as fibronectin (FN) and integrins, providing a favorable tumor microenvironment (11, 12). On the cell surface, the interaction between FN and integrin β1 activates a complex network of ‘outside-in’ signaling, including engagement of focal adhesion (FA) complexes and subsequent activation of downstream signaling effectors phosphatidylinositol-3-kinase (PI3K)/protein kinase B (AKT) and mitogen-activated protein kinases (MAPKs). These signaling pathways have been shown to modulate the oncogenic Wnt/β-catenin, NOTCH, and Hedgehog pathways, which are associated with stemness phenotypes, including resistance to chemotherapy (12–14).

Integrin-linked kinase (ILK), a serine/threonine protein kinase, plays unifying role in the “outside-in” transduction cascade (15). ILK binds to a broad range of adaptor and signaling molecules, such as the cytoplasmic domains of integrin β1, β3 or β5 subunits (16), PINCH (particularly interesting new cysteine–histidine rich protein), and parvin (17). These interactions modulate ILK phosphorylation (18, 19) to regulate response to growth factors and secreted glycoproteins (18, 19). Active ILK modulates downstream effector proteins such as AKT and glycogen synthase kinase-3α/β (GSK-3α/β) (20, 21), and aberrant ILK activation has been linked to anoikis resistance by promoting AKT phosphorylation and activation of pro-survival pathways through up-regulation of the anti-apoptotic B-cell lymphoma 2 (Bcl-2) (21). Previously, we demonstrated that secretion of FN activated ILK and inhibitory phosphorylation of GSK-3α/β at Ser^21/9^, resulting in amplification of β-catenin signaling and promotion of OC cell proliferation and migration (6, 12). Thus, a direct involvement of ILK as a modulator of the crosstalk between ECM re-arrangement and cell survival and growth has been suggested but a role for ILK in the platinum tolerant CSC phenotype has not been investigated.

We recently demonstrated that targeting FN/integrin β1 inhibited Wnt/β-catenin, ALDH1A1 and OC sphere formation, sensitized OC cells to platinum and inhibited tumorigenesis (6, 12, 22). Here, we show that OC spheroids maintain a high level of active ILK (p-ILK^Ser246^), consistent with aberrant FN secretion and integrin β1 expression (12). Expression of ILK was increased in OCSCs (ALDH^+^/CD133^+^) and increased ILK levels correlated with ALDH1A1 expression and poor patient outcome. Mechanistically, by interacting with the Wnt receptor frizzled 7 (Fzd7), ILK activated “outside in” signaling, resulting in amplified Wnt-3A signals, sustained β-catenin-TCF/LEF1 transcriptional activity, and increased spheroid formation. Combined inhibition of both Fzd7 and ILK plus carboplatin blocked β-catenin nuclear translocation, inhibited AKT phosphorylation at Ser473, and increased a pro-apoptotic cascade, resulting in the elimination of platinum resistant OCSCs. We suggest a therapeutic strategy based on targeting ECM components or their key downstream elements to eliminate OCSCs.

## Materials and Methods

### Chemicals and reagents

Unless stated otherwise, chemicals and reagents were from Sigma-Aldrich (St Louis, MO, USA). The antibodies used are listed in supplementary Table S1. Recombinant human Wnt-3A was purchased from R&D Systems (Minneapolis, MN). ILK inhibitor, cpd-22 was purchased from Millipore-Sigma (Burlington, MA, USA).

### Cell lines

OVCAR-3 cell line was from the American Type Culture Collection (Rockville, MD). COV362, HEY-A8, PEA1, PEA2 cells were provided by Dr. Yan Xu (Indiana University). Cell lines were authenticated by Short Tandem Repeat (STR) analysis and tested to be mycoplasma negative by IDEXX (BioResearch, Columbia, MO). All cells were cultured in D-MEM (high glucose), 10% FBS, 2 mM L-glutamine, and 100 units/mL penicillin and 100 μg/mL streptomycin. Cells were cultured at 37°C in a humidified incubator with 5% CO2 supply.

### Primary human cells

De-identified malignant ascites fluid specimens from OC patients (n = 4) were obtained at the Indiana University Simon Comprehensive Cancer Center (IUSCCC) under an IRB approved protocol (HRPP #1905951308). All subjects had stage 3 or 4 HGSOC or primary peritoneal carcinomatosis. For primary cells isolation from human specimens (OC ascites and primary tumors), tumor cells were collected and purified as previously described (6).

### Spheroid proliferation

OC cells were resuspended in Mammocult complete media and seeded into ultra-low adherence 96-well plates (2.5 × 10^3^ cells/well) as previously described (6, 12). Proliferation was quantified by the CCK-8 assay (Dojindo Molecular Technologies, Rockville, MD). Optical density of the amount of the formazan dye was measured with a plate reader (800TS Absorbance Reader, BioTek) at 450 nm. Three replicates were used and data are means ± SD.

### Colony formation

OC cells were seeded into 6-well plates (500 cells/well) and allowed to attach overnight. Cells were exposed to the appropriate treatment and cultured for 10–14 days to form colonies. The debris was washed twice with 1 ml of PBS, and the cells fixed with 4% paraformaldehyde in PBS and stained with 0.05% crystal violet for counting. Three replicates were used and data are means ± SD.

### ALDEFLUOR assay and Fluorescence-Activated Cell Sorting

Dissociated OC single cells were centrifuged at 1,500 rpm for 5 min, resuspended in Aldefluor assay buffer (Stemcell Technologies) containing the ALDH substrate, bodipyaminoacetaldehyde (BAAA), at 1.5mM as previously described (6, 12). FACS was performed using a FACSAria II flow cytometer (BD Biosciences, San Jose, CA) under sterile conditions.

### Development of carboplatin resistant OC cells

Carboplatin resistance was established by deriving carboplatin-resistant OVCAR3 and HEY-A8 cells from the correspondent parental cells through continuous exposure to carboplatin (Sigma-Aldrich). OC cells were subjected to initial dose-response studies of carboplatin (1–100 μM) over 72 h to determine IC_50_ values. The media was removed and surviving cells were allowed to recover till reached 70–80% confluence. The drug regimen was continued in the same manner for approximately 4–6 cycles. Subsequently, the IC_50_ concentrations were re-evaluated in the resistant OVCAR3 and HEY-A8 cells. The cells were then continuously maintained in the presence of carboplatin at these updated IC_50_ concentrations for an additional 4–6 cycles (23).

### Half maximal inhibitory concentration (IC_50_)

The IC_50_ values of carboplatin was determined by using CCK-8 proliferation assay kit. IC_50_ values were determined by logarithm-normalized sigmoidal dose curve fitting using Prism 10 software (GraphPad Software Inc., San Diego, CA) (24).

### Transfection

Stable gene knockdown was obtained by using lentiviral transduction particles containing ShRNA targeting ILK (sh-ILK), Fzd7 (sh-Fzd7) or scrambled ShRNA control (Sigma-Aldrich,) into HEY-A8 and/or OVCAR-3 cells (12, 22). Lentiviral-transduced OC cells were selected with puromycin (1.5 μg/ml). Transient transfection used a pool of 4 short interfering RNAs (siRNA) targeting β-catenin (Dharmacon, Pittsburgh, PA, USA) or individual siRNA sequences and DreamFECT transfection reagent (Oz Biosciences, Marseille, France). Scrambled siRNA pool (Dharmacon) was used as control (6).

### Quantitative real-time PCR (qRT-PCR)

Total RNA was extracted using RNA STAT-60 Reagent (Tel-Test Inc., Friendswood, TX) and one μg was reverse-transcribed using iScript cDNA synthesis kit (Bio-Rad, Hercules, CA, USA). Amplification of cDNA was performed by quantitative RT-PCR on an ABI Prism 7500 platform (Applied Biosystems). The primers used are listed in Supplementary Table S2. Results are presented as means +/− SD of replicates. Each experiment was performed in duplicate in three independent conditions.

### Chromatin immunoprecipitation (ChIP) assay

ChIP assay was performed in OC spheroids using a kit from Millipore as described previously (12). PCR amplification was performed using primers designed for the β-catenin/TCF/LEF1 binding domain of the ILK and c-Myc promoters (Table S3 Supplementary data). As a negative control, DNA immunoprecipitated with β-catenin antibody was amplified with primers designed for the ILK promoter upstream of the TCF/LEF1 binding site non-target region (NTR).

### Co-immunoprecipitation (Co-IP):

Total lysates from OC cells grown as spheroids were centrifuged at 1,500 rpm for 5 min, washed in PBS (phosphate buffered saline 1X) and lysed on ice IP lysis buffer (Thermo Scientific Pierce, Waltham, MA USA). In parallel experiments, recombinant human full length Fzd7 was mixed in lysis buffer at 1:1 ratio with recombinant human full-length ILK. Mixtures of cell lysates or recombinant proteins were incubated overnight at 4° C with anti-Fzd7, anti-ILK, or IgG respectively and processed as described previously (12, 22).

### WB analysis

Equal amounts of protein were separated by SDS-PAGE and electroblotted onto PVDF membranes. Images were captured by a luminescent image analyzer with a CCD camera (Chemi-Doc Imaging System, Bio-Rad) and quantified by densitometric analysis with a Gel-Pro Analyzer 3.1 software. All proteins were normalized with the structural protein GAPDH levels. Phosphorylated proteins were also normalized with their total pair. Protein levels were expressed as average value and presented in the graphs as means ± SD (N = 3).

### Immunofluorescence (IF):

Patient-derived OC primary spheroids were collected by centrifugation at 1,500 rpm for 5 min at 4°C, washed 3 X with cold PBS, and fixed, permeabilized, and stained following a previously described protocol (6, 12). The corrected total cell fluorescence (CTCF) was calculated by using the following formula: CTCF = Integrated Density − (Area of selected cell × Mean fluorescence of background readings). Quantification of co-localized proteins was calculated by using co-localization test macro in ImageJ software. For the negative control experiments, only the anti-rabbit or -mouse IgG isotypes were incubated with the Alexa fluor-647- or Alexa fluor-488-conjugated anti-rabbit or anti-mouse secondary antibodies. Each image is representative of at least three biological replicates.

### In situ proximity ligation assay (PLA)

Interaction between Fzd7 with p-ILK^Ser246^ were measured in patient-derived OC primary cells by PLA using Duolink reagents (Millipore-Sigma) and following the manufacturer recommendations as described previously (12, 22). Spheroids were observed with a confocal/two-photon Olympus Fluoview FV-1000 MPE system. Fluorescence was quantified using the analyze particles macro in ImageJ software. For every antibody, a negative control experiment was performed where only one antibody was incubated with the PLA probes. Each image is representative of at least three biological replicates.

### Immunohistochemistry (IHC):

De-identified human HGSOC specimens (stage: 3 + 4, grade: 3) (88 total tissues) on a tissue microarray (TMA) were obtained from the Biospecimen Collection and Banking Core at IUSCCC under an IRB approved protocol (HRPP #1905951308). The OC-TMA includes paired samples of normal ovary and fallopian tube epithelium, primary tumors from patients diagnosed with low and high-grade serous (stage: 3 + 4, grade: 3) ovarian carcinoma, and metastatic specimens. An H score was calculated as the product between intensity and percentage of stained cells and tumors were classified as positive or negative if H score was > or < median score, respectively.

### In vivo subcutaneous xenograft mouse model

All animal studies were approved by the Institutional Animal Care and Use Committee at Indiana University School of Medicine and were in compliance with the The Animal Care and Use Review Office of the USAMRDC Office of Research Protections (ORP). FACS-isolated ALDH^+^/CD133^+^ HEY-A8 cells were resuspended in Mammocult complete medium (StemCell Technologies) and seeded at a density of 10,000 cells/well in non-adherent ultra-low plates. An equal number of DMSO control and cpd-22 treated spheres were injected subcutaneously into the left and right flank of 5–6 week-old female NSG mice, respectively, with 4 mice randomly assigned to each group. Tumor volume was calculated as 1/2 × L × W2, where L stands for the length, and W for the width measured by a caliper in mm. At the end of the study (e.g., when at least one tumor reached 2,000 mm3), mice were euthanized, tumors were harvested, measured and weighed.

### In vivo intraperitoneal xenograft mouse model:

5–6 week-old female NSG mice were divided into 4 groups: 1) DMSO, 2) cpd-22, 3) DMSO + carboplatin, and 4) cpd-22 + carboplatin, with 5 mice randomly assigned per group. Mice were weighed and HEY-A8 cells were injected intraperitoneally at a density of 5 Χ 10^6 cells/mouse. Mice were treated with cpd-22 (25 mg/kg by oral gavage, two times a week) or combination with carboplatin (25 mg/kg two times a week), beginning one week after ip xenograft. At the end of the study, mice were weighed and euthanized. Tumors were harvested, measured and weighed, and metastasis counted. Tumor volume was calculated as 1/2 × L × W2.

### Analysis of TCGA ovarian cancer cohort:

The RNASeqv2 level 3 data for the TCGA ovarian cancer samples and the clinical information associated with these samples were obtained from cBioPortal (http://www.cbioportal.org). Survival analysis was performed in advanced HGSOC patients (stage: 3 + 4, grade: 3) using Kaplan-Meier plotter (KM) (25) or OvMark system (26) and the statistical significance was defined as log-rank P value < 0.05. KM plots for patients with no residual tumors and *ALDH1A1, ILK*, and/or *Fzd7* median expression, high (within the 75% quartile) or low expression (within 25% quartile), were used as the cutoff. The pan-cancer analysis displayed differences in the *m*RNA expression profile of *ILK* between OC and metastatic tissue specimens evaluated with the non-parametric Wilcoxon Signed Ranks test. OC tissues were classified according to their clinico-pathological features (TNM stage, lymph node invasion status) and statistical analysis was performed with the use of Mann-Whitney U test. Patients diagnosed with advanced serous OC (stage: 3, grade: 3) and received neoadjuvant chemotherapy were classified according to pathological response, no residual histological evidence of the tumor remains after chemotherapy (responders) and all other patients with residual tumor tissue (non-responders). The two cohorts were compared using Mann–Whitney test or Receiver Operating Characteristic (ROC) test. Statistical analysis was performed using GraphPad Prism 9 software.

### Statistical Analysis

Data were analyzed using one-way ANOVA followed by Tukey’s post-hoc test or unpaired two-tailed t-test. Results are represented as means ± SD, with *P < 0.05 considered statistically significant. Each experiment was performed with a minimum of three biological replicates; exact numbers are indicated in associated figure legends.

## Results

### ILK correlates with HGSOC patient outcomes and OCSC phenotypes.

**Figure 1:**
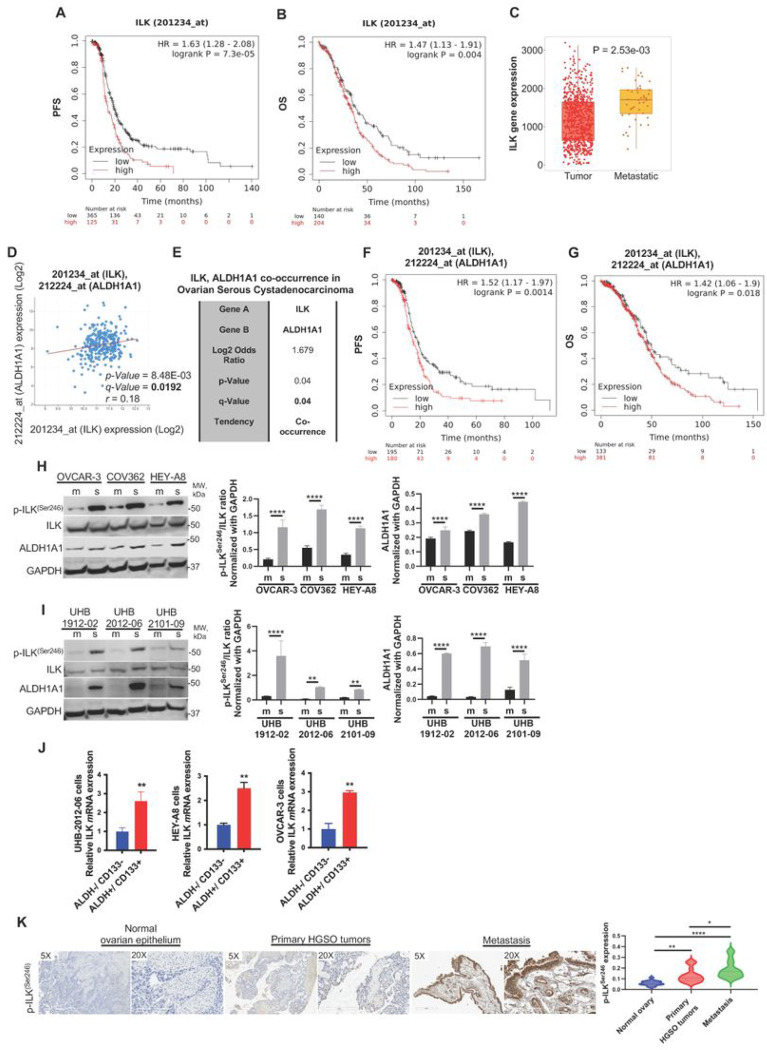
ILK promotes the CSC phenotype and correlates with poor PFS and metastasis in OC patients. **A.** PFS curves for *ILK m*RNA expression in HGSOC patients generated using the Kaplan–Meier plotter (HR = 1.63 and P = 7.3e-05). **B**. OS curves for *ILK m*RNA expression in HGSOC patients generated using the KM plotter (HR = 1.47 and P = 0.004). **C.** TNMplot showing a significant increase of ILK expression in metastatic samples compared to primary tumor (P = 2.53e-03). **D.** Correlation between *ALDH1A1* and *ILK m*RNA expression levels (Spearman r = 0.18, P = 8.48e-03, Q = 0.0192) in the OC TCGA datasets obtained from cBioPortal. **E**. *ALDH1A1* and *ILK m*RNA expression levels show a strong tendency toward co-occurrence (Log2 odds ratio = 1.679, P = 0.04, Q = 0.04) in the OC TCGA datasets obtained from cBioPortal. **F**. PFS curves for *ALDH1A1* and *ILK m*RNA expression in HGSOC patients generated using the KM plotter (HR = 1.52 and P = 0.0014). **G**. OS curves for *ALDH1A1* and *ILK m*RNA expression in HGSOC patients generated using the Kaplan–Meier plotter (HR = 1.42 and P = 0.018). **H**. WB for p-ILK^Ser246^, ILK, ALDH1A1, and GAPDH in OVCAR-3, COV362, and HEY-A8 monolayers (m) vs. spheroids (s) (left panel). Densitometry quantifies ALDH1A1 and p-ILK^Ser246^/ILK ratio (right panel) (N = 3; ****P < 0.0001). **I**. WB for p-ILK^Ser246^, ILK, ALDH1A1, and GAPDH in UHB-1912-02, UHB-2012-06, and UHB-2101-09 primary OC cells grown as spheroids vs. monolayers (m) (left panel). Densitometry quantifies ALDH1A1 and p-ILK^Ser246^/ILK ratio (right panel) (N = 3; **P < 0.01 and ****P < 0.0001). **J**. *ILK m*RNA levels measured by qRT-PCR in ALDH^+^/CD133^+^ vs ALDH^−^/CD133^−^ isolated from UHB-2012-06 primary human OC cells, HEY-A8, and OVCAR-3 cells (N = 3; **P < 0.01). **K**. P-ILK^Ser246^ expression levels detected by IHC in human serous ovarian tumors, metastatic, and normal ovarian epithelium included on a TMA (left). Representative images are shown (magnification, × 5 and × 20). Quantification of p-ILK^Ser246^ protein levels by intensity with H-score in OC TMA analysis (right). Error bars show mean (±STD) (N = 3; *P < 0.05, **P < 0.01, ****P < 0.0001).

Analysis of survival curves of advanced HGSOC patients (stage: 3 + 4, grade: 3) in KM database demonstrated that higher than median *ILK* expression correlated with poor PFS and OS after optimal debulking surgery (Fig. 1A,B, respectively), In addition, *ILK* expression was higher in HGSOC metastatic samples compared to primary tumor (TNMplot analysis; Fig. 1C), further supporting a role of ILK in OC progression. Regression analysis of HGSOC tumors in the TCGA demonstrated that *ILK* and *ALDH1A1* levels were highly correlated (Fig. 1D), and co-occurring alterations between *ILK* and *ALDH1A1* were observed (Fig. 1E). Moreover, higher than median *ILK* and *ALDH1A1* combined expression levels were associated with worse progression-free survival (PFS) and overall survival (OS) after optimal debulking surgery in patients with advanced HGSOC (stage: 3 + 4, grade: 3) (Fig. 1F,G, respectively). Based on these strong correlations, we further examined ILK in OCSC phenotypes.

We measured p-ILK^Ser246^ in human HGSOC cell lines and primary cells isolated from malignant ascites of HGSOC patients. Increased expression of p-ILK^Ser246^ and ALDH1A1 was observed in OC cell lines and primary cells grown as spheroids compared to monolayers (Fig. 1H, I), as was increased expression of stemness-related markers *ALDH1A1, Sox-2*, *Nanog* and *Oct-4* (Fig. S1A). Furthermore, expression of ILK (Fig. 1J) and stemness markers (Fig. S1B) was increased in OCSCs (ALDH^+^/CD133^+^) compared to non-OCSCs (ALDH^−^/CD133^−^). In primary tumors from patients diagnosed with HGSOC (stage: 3 + 4, grade: 3), p-ILK^Ser246^ was greater compared to corresponding adjacent ovarian epithelium (Fig. 1H), demonstrating increased ILK activation, and p-ILK^Ser246^ staining was greatest in metastatic tumors (Fig. 1K and Table S4). Taken together, these results support further examining ILK in OC progression and chemoresistance.

### ILK inhibition inhibits OCSCs in vitro and prevents tumor-initiating capacity in vivo.

**Figure 2:**
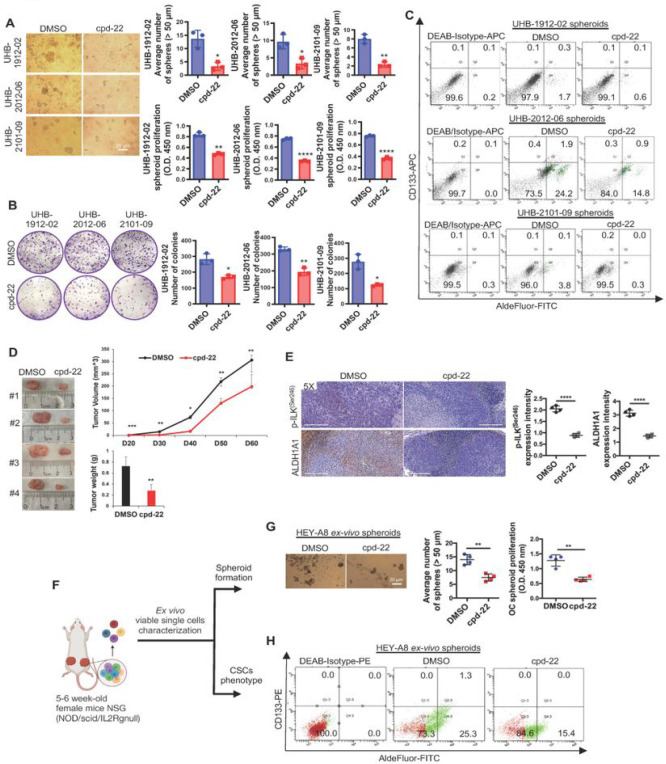
ILK inhibition blocks OC spheroid proliferation, colony formation, and tumor initiation. **A.** Representative phase contrast images of three different HGSOC primary cells (UHB1912-02, UHB2012-06, and UHB2101-09) growing as spheroids and treated with cpd-22 (left panel). Average number of spheroid count (> 50 μm in diameter) (upper right panel) and CCK-8 quantification (lower right panel) of HGSOC primary cells treated with DMSO control vs cpd-22 (N = 3; *P < 0.05, **P < 0.01, ****P < 0.0001). **B**. Clonogenic assay of HGSOC primary cells treated for 7 days with DMSO control or cpd-22 (0.5 μg/ml). Representative images of single-cell clone proliferation, stained with crystal violet (left panel). Quantification of the results (right panel) (N = 3; *P < 0.05 and ***P < 0.001P). **C**. Flow cytometry measures ALDEFLUOR-FITC+/CD133-APC+ cells in HGSOC primary cells (UHB1912-02, UHB2012-06, and UHB2101-09) growing as spheroids and treated with DMSO control or cpd-22. DEAB/APC-Isotype treated cells serve as negative controls. Measurements were performed in three replicates. **D**. Tumor morphology (left panel), tumor volumes (upper right panel), and weights (lower right panel) derived from ALDH^+^/CD133^+^ sorted from HEY-A8 cells and treated with cpd-22 or DMSO control and injected sc in NSG mice, as described (N = 5; *P < 0.05, **P< 0.01, ***P < 0.001). **E**. IHC analysis of fixed tumor sections prepared from sc implants. Quantification of p-ILK^Ser246^ and ALDH1A1 protein levels by intensity with Gleason score. Error bars show mean (±STD) (N = 3; ****P < 0.0001). **F**. Graphical representation of ex vivo single cell characterization. **G**. Representative spheroid morphology (left panel) and proliferation assay (right panel) of cells isolated from xenografts and grown ex vivo. (N = 3; **P < 0.01). **H**. Flow cytometry measures ALDEFLUOR-FITC+/CD133-PE+ cells in ex vivo OC cells isolated from xenograft and grown as spheroids. DEAB/APC-Isotype treated cells serve as negative controls. Measurements were performed in three replicates.

To examine the functional role of ILK activation in OCSCs, ILK expression levels or activity were altered by using either sh-RNA mediated knockdown (KD) or pharmacological inhibition with compound 22 (cpd-22; specific cell-permeable ILK inhibitor (27)). ILK expression levels (Fig. S2A), spheroid formation (Fig. S2B) and colony formation capacity (Fig. S2C) were significantly decreased in sh-ILK transduced OC cells. Treatment of HGSOC primary cells with cpd-22 inhibited both spheroid and colony formation by (Fig. 2A,B), and flow cytometry analysis showed that cpd-22 treatment decreased the ALDH^+^/CD133^+^ population in HGSOC primary spheroids compared to DMSO vehicle control (Fig. 2C).

OCSCs are responsible for initiating tumor formation in vivo when injected in immunocompromised mice (31). ALDH^+^/CD133^+^ cells (1 × 10^5 cells) were cultured under stem cell conditions and treated with cpd-22 or DMSO for 7 days and then injected SC into the flanks of female nude mice. Tumor growth was inhibited by cpd-22 (Fig. 2D), and active-p-ILK^Ser246^ and ALDH1A1 levels in the tumors were decreased by treatment with cpd-22 (Fig. 2E). In addition, single cells derived from cpd-22-treated tumors (Fig. 2F) were incapable of forming spheroids (Fig. 2G) and the ALDH^+^/CD133^+^ cell population was reduced compared to cells derived from vehicle treated tumors (Fig. 2H). These results support a key role for p-ILK^Ser246^ activation in maintaining the OCSC phenotype and ILK as target for reducing OC tumor formation.

### ILK modulates Fzd7 expression to maintain the OCSC phenotype.

**Figure 3:**
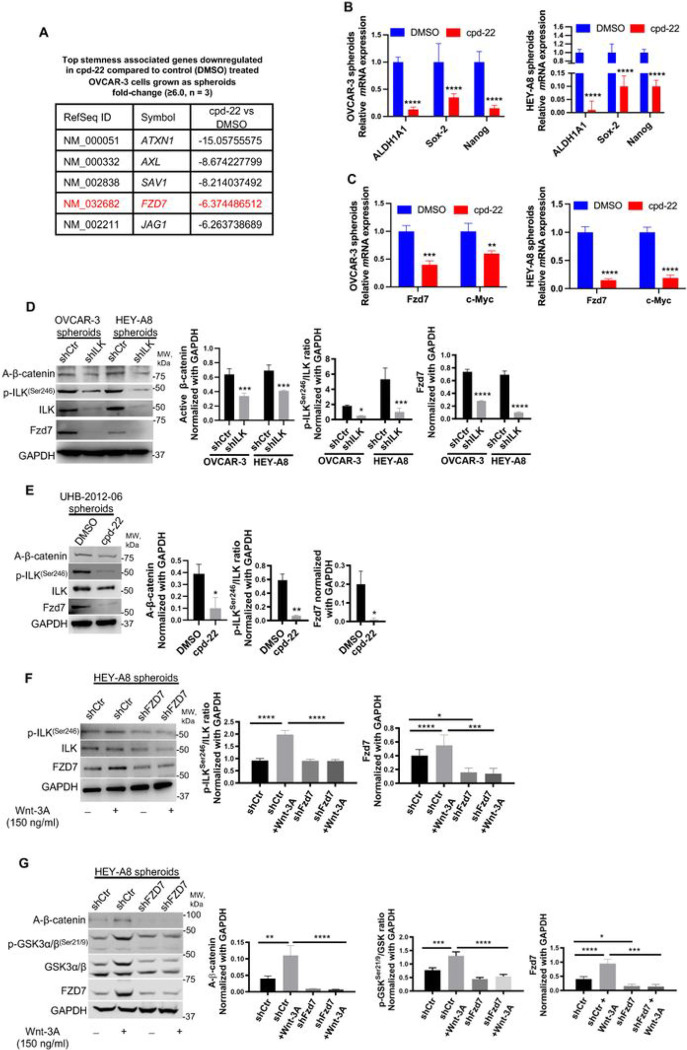
ILK inhibition blocks the OCSC phenotype and regulates Fzd7 expression. **A.** CSC genes down-regulated in OVCAR-3 spheroids by cpd-22 treatment compared to DMSO control (≥2.0-fold change). **B.** *ALDH1A1*, *Sox-2*, and *Nanog m*RNA levels measured by qRT-PCR in OVCAR-3 and HEY-A8 spheroids treated with DMSO control or cpd-22 (N = 3; ****P < 0.0001). **C**. QRT-PCR for *Fzd7* (left panel) and *c-Myc* (right panel) *m*RNA levels in OVCAR-3 and HEY-A8 spheroids treated with cpd-22 vs. DMSO (N = 3; **P < 0.01, ***P< 0.001, ****P < 0.0001). **D**. WB for A-β-catenin, Fzd7, p-ILK^Ser246^, ILK, and GAPDH in OVCAR-3 and HEY-A8 spheroids shCtr and shILK (clone #3) (left panel). Densitometry quantifies A-β-catenin and Fzd7 expression levels and p-ILK^Ser246^/ILK ratio (N = 3; *P < 0.05, ***P < 0.001, ****P < 0.0001). **E**. WB for A-β-catenin, Fzd7, p-ILK^Ser246^, ILK, and GAPDH in UHB-2012-06 HGSOC primary spheroids treated with DMSO or cpd-22 (left panel). Densitometry quantifies A-β-catenin and Fzd7 expression levels and p-ILK^Ser246^/ILK ratio (N = 3; *P < 0.05 and **P < 0.01). **F**. WB for p-ILK^Ser246^, ILK, Fzd7, and GAPDH in HEY-A8 shCtr and shFzd7 (clone #2) grown as spheroids and treated or not for 7 days with Wnt-3A (left panel). Densitometry quantifies Fzd7 expression levels and p-ILK^Ser246^/ILK ratio (right panel) (N = 3; *P < 0.05, ***P < 0.001, ****P < 0.0001). **G**. WB for A-β-catenin, p-GSK-3α/β^Ser21/9^, GSK-3α/β, Fzd7, and GAPDH in HEY-A8 shCtr and shFzd7 (clone #2) grown as spheroids and treated or not for 7 days with Wnt-3A (left panel). Densitometry quantifies A-β-catenin and Fzd7 expression levels and p-GSK-3α/β^Ser21/9^/GSK-3α/β ratio (right panel) (N = 3; *P< 0.05, **P < 0.01, ****P < 0.0001).

To further examine the underlying mechanism of action of ILK in OCSCs, FACS-sorted ALDH^+^/CD133^+^ cells were isolated, treated with cpd-22, grown as spheroids for 7 days and evaluated using a human CSC-focused gene array. Expression of genes related CSC maintenance (*ALDH1A1, KIT, Prom1*) and pluripotency (*Myc, Nanog, Sox-2*) was downregulated in cpd-22-treated cells compared to control cells (Fig. 3A, B; Table S5). Furthermore, we examined a key developmental pathway linked to cancer stemness in OC spheroids, the Wnt pathway, and ILK inhibition markedly downregulated several pathway members, including *Fzd7* and β-catenin target gene *c-Myc* at *m*RNA levels (Fig. 3A, C; Table S4). WB of ILK-KD OC cells grown as spheroids showed decreased p-ILK^Ser246^, Fzd7 and non-p-(active) β-Catenin^Ser33/37/Thr41^ (A-β-catenin) expression levels compared to shCtr cells (Fig. 3D). Consistently, p-ILK^Ser246^, Fzd7 and A-β-catenin levels were reduced in HGSOC primary spheroids treated with cpd-22 (Fig. 3E). Moreover, Wnt-3A-treatment of OC spheroids with Fzd7-KD decreased p-ILK^Ser246^ levels (Fig. 3F, Fig. S3B) and phosphorylation of GSK-3α/β at Ser21/9 was abrogated, preventing nuclear translocation of A-β-catenin spheres (Fig. 3G, Fig. S3C). Increased Fzd7 expression levels in the presence of Wnt-3A (Fig. 3F,G) confirmed direct β-catenin transcriptional regulation of this Wnt receptor (28).

### ILK forms a complex and directly interacts with Fzd7 in OC spheroids.

**Figure 4:**
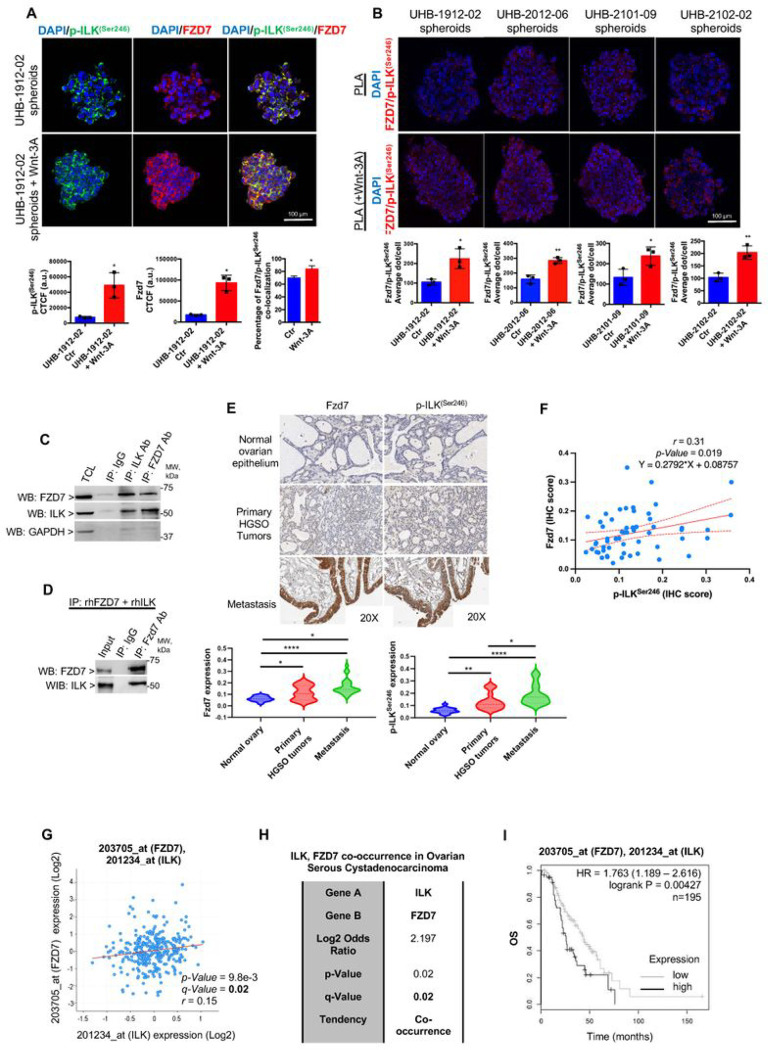
ILK functionally correlates with Fzd7 in OC spheroids and tumors. **A.** IF staining for p-ILK^Ser246^ (Alexa Fluor 488, green) and Fzd7 (Alexa Fluor 647, red) in UHB-1912-02 primary OC cells grown as spheroids and treated or not with Wnt-3A. Protein co-localization is identified by yellow spectra on merged images (upper panel). Quantification of Alexa Fluor 488 (green) and Alexa Fluor 647 (red) proteins was calculated by using Metamorph software (N = 3; *P < 0.05). Quantification of co-localized proteins was calculated by volume area of green over red spectra by using Metamorph software (N ≥ 3; *P < 0.05). **B**. Fzd7/p-ILK^Ser246^ colocalization (red dots) and phalloidin (green) detected by PLA in primary human cells. Representative images are shown (magnification, × 200). Bar, 20 μm. Quantification of the number of total Fzd7/p-ILK^Ser246^ red dots per sample in a diagram (N = 3; *P < 0.05, **P < 0.01). **C**. Equal amounts of lysates of HEY-A8 cells grown as spheroids were immunoprecipitated with ILK and Fzd7 Abs. WB was performed using ILK, Fzd7, and GAPDH Abs. **D**. Co-IP with anti-Fzd7 Ab and WB for ILK and Fzd7 using full-length recombinant ILK and Fzd7. **E**. Fzd7 and p-ILK^Ser246^ expression levels detected by IHC in human ovarian tumors, metastatic, and normal surface ovarian epithelium specimens included on a TMA (left. Representative images are shown (magnification, × 20). Quantification of Fzd7 and p-ILK^Ser246^ protein levels by H intensity in OC TMA analysis (right). Error bars show mean (±STD) (N = 3; *P < 0.05, **P < 0.01, ***P < 0.001, and ****P < 0.0001). **F**. Correlation between Fzd7 and p-ILK^Ser246^ protein levels (Pearson r = 0.31, P = 0.019). **G**. Correlation between *ILK* and *Fzd7 m*RNA expression levels (Spearman r = 0.15, P = 9.8e-03 and Q = 0.02) in the TCGA OC database obtained from cBioPortal. **H**. *Fzd7* and *ILK m*RNA expression levels show a strong tendency toward co-occurrence (Log2 odds ratio = 2.197, P = 0.02, Q = 0.02) in the OC TCGA datasets obtained from cBioPortal. **I**. OS curves generated using HGSOC tumor microarray data of 14 datasets from 7 different array platforms using OvMark for tumors expressing higher than median levels of *ILK* and of *Fzd7* versus those expressing lower than median levels of *ILK* and of *Fzd7* (HR = 1.763 and P = 0.00427).

As active ILK may act as a Wnt/β-catenin co-activator through Fzd7, it was of interest to examine a possible interaction between ILK and Fzd7. First, OC spheroids isolated from two primary OC patient specimens and treated with Wnt-3A showed increased IF staining for Fzd7 and p-ILK^Ser246^ compared to untreated spheroids (Fig. 4A). IF confocal and co-localization analysis showed that Fzd7 and p-ILK^Ser246^ co-localized in HGSOC primary spheroids and that co-localization increased in the presence of Wnt-3A (Rcoloc = 0. 6, Fig. 4A). Second, to determine whether Fzd7-p-ILK^Ser246^ complexes were detectable in human tumors, proximity ligation assay (PLA), a technique capable of identifying proteins localized within 40 nm distance in tissue, was utilized. Fzd7-p-ILK^Ser246^ complex formation was detectable in spheroids derived from primary human malignant ascites and increased upon Wnt-3A treatment (Fig. 4B). Next, co-IP in cell lysates from OC spheroids and human recombinant Fzd7 and ILK proteins demonstrated that Fzd7 and ILK were detectable in complexes immunoprecipitated with anti-ILK and anti-Fzd7 Abs (Fig. 4C,D).

It was also of interest to evaluate OC patient specimens for a Fzd7 and p-ILK^Ser246^ correlation. IHC analysis in primary tumors from patients diagnosed with HGSOC (stage: 3 + 4, grade: 3) revealed increased Fzd7 and active-p-ILK^Ser246^ levels compared to corresponding adjacent normal ovarian epithelium (Fig. 4E), both Fzd7 and p-ILK^Ser246^ staining was greatest in metastatic tumors (Fig. 4E and Tables S4 and S6). A regression analysis further revealed a high correlation between active ILK and Fzd7 levels in patient tumors (Fig. 4F; Pearson r = 0.31, P = 0.019), and a tendency of co-occurring alterations between *ILK* and *Fzd7* in HGSOC tumors in the TCGA database (Fig. 4G,H). Furthermore, analysis of HGSOC tumor microarray data using OvMark demonstrated an increased estimated risk of death in patients with higher than median *Fzd7* and *ILK* combined expression levels compared with patients with lower than median *Fzd7* and *ILK* expression levels (Fig. 4I). Overall, the results support ILK, through its Fzd7 partner, as a direct Wnt/β-catenin co-activator and plays a key role in OC spheroid formation and OC metastasis.

### ILK is a β-catenin target gene.

Based on the observation that ILK was activated by Wnt-3A treatment (Fig. 3F), it was of interest to examine whether ILK is a direct Wnt target gene. As shown in Fig. S4A, ILK expression was decreased by siRNA-mediated β-catenin downregulation, supporting the hypothesis that ILK is transcriptionally regulated by β-catenin. By using a promoter motif searching software (PROMO), a potential TCF/LEF-responsive element was identified in the ILK promoter sequence (positions − 103 to − 96). To determine whether β-catenin interacts directly with the ILK promoter, chromatin pulled down by a β-catenin antibody were PCR amplified using primers specific for fragments corresponding to the TCF/LEF-responsive regions (Fig. S4B). No PCR product in chromatin immunoprecipitated with immunoglobulin G (IgG) was observed (Fig. S4B), demonstrating the specificity of the β-catenin antibody used for ChIP and that ILK is a direct Wnt/β-catenin target in OC cells.

### ILK/Fzd7 predict response to chemotherapy and its targeting sensitizes OC to platinum.

**Figure 5:**
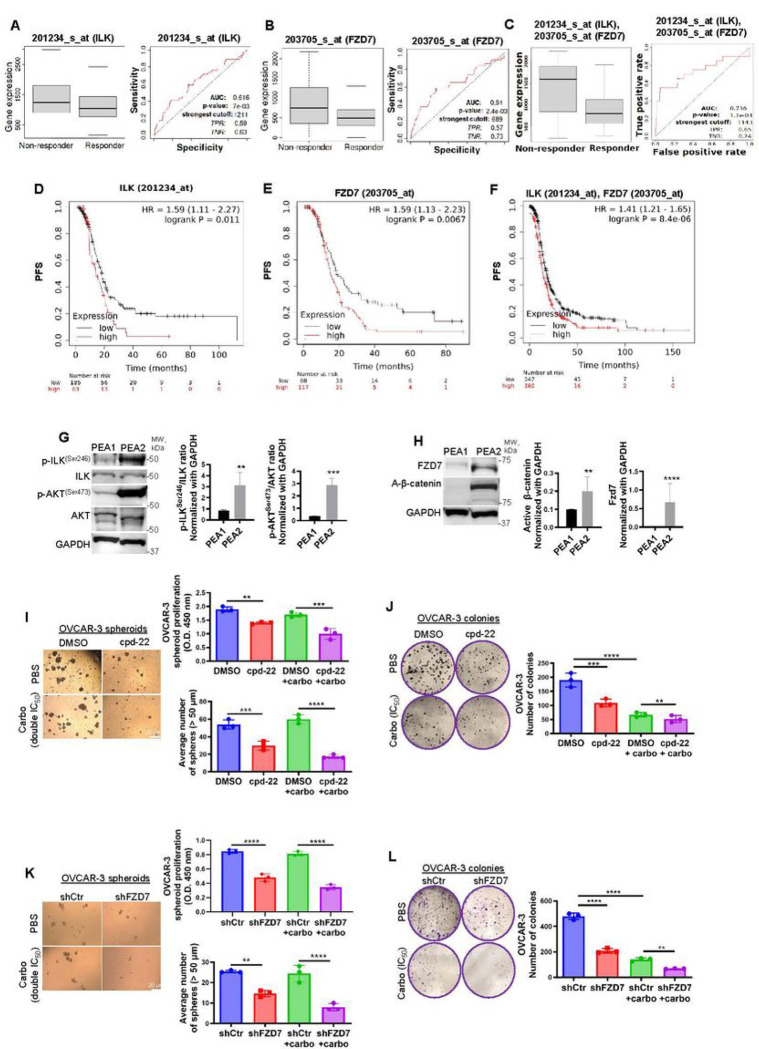
Fzd7-KD and ILK inhibition sensitize to platinum treatment. **A-C.** Boxplot and ROC curve of ILK (**A**), *Fzd7* (**B**), and *ILK-Fzd7* combination (**C**) involved in platinum resistance using the RFS at 6 months cohort from the ROC plotter for OC (Affymetrix ID: 201234_s_at (ILK) and 203705_s_at (Fzd7), respectively). ROC curve analysis shows the sensitivity and specificity of *ILK* and *Fzd7* in predicting the patient response to treatment (P = 7e-03, P = 2.4e-03, and P = 1.1e-03, respectively). **D-F**. PFS curves for *ILK* (**D**), *Fzd7* (**E**), and *ILK* in combination with *Fzd7* (**F**) mRNA expression in HGSOC patients (stage: 3 + 4, grade: 3) following platinum/taxane therapy generated using the KM plotter (HR = 1.59, P = 0.011; HR = 1.59, P = 0.0067; and HR = 1.41, P = 8.4e-06, respectively). **G**. WB for p-ILK^Ser246^, ILK, p-AKT^Ser473^, AKT, and GAPDH in HGSOC platinum (S) PEA1 and platinum (R) PEA2 cells (left panel). Densitometry quantifies p-ILK^Ser246^/ILK and p-AKT^Ser473^/AKT ratio (right panel) (N = 3; **P < 0.01 and ***P < 0.001). **H**. WB for A-β-catenin, Fzd7, and GAPDH in PEA1 and PEA2 cells (left panel). Densitometry quantifies A-β-catenin and Fzd7 expression levels (right panel) (N = 3; **P < 0.01 and ****P < 0.0001). **I**. Representative phase contrast images of OVCAR-3 cells growing as spheroids and treated with DMSO, carboplatin (double IC_50_), cpd-22 or combination (right panel). CCK-8 quantification (upper-left panel) and average number of spheroid count (> 50 μm in diameter) (lower-left panel) (N = 3; **P < 0.01, ***P < 0.001, ****P < 0.0001). **J**. Clonogenic assay of OVCAR-3 cells treated with DMSO, carboplatin (IC_50_), cpd-22 or combination. Representative images of single-cell clone proliferation, stained with crystal violet (right panel) and quantification (left panel) (N = 3; **P < 0.01, ***P < 0.001, ****P < 0.0001). **K**. Representative phase contrast images of shCtr vs shFzd7 (clone #2) OVCAR-3 cells growing as spheroids and treated with carboplatin (double IC_50_) (right panel). CCK-8 quantification (upper-left panel) and average number of spheroid count (> 50 μm in diameter) (lower-left panel) (N = 3; **P < 0.01 and ****P < 0.0001). **L**. Clonogenic assay of shCtr vs shFzd7 (clone #2) OVCAR-3 cells treated with carboplatin (IC_50_). Representative images of single-cell clone proliferation, stained with crystal violet (right panel) and quantification (left panel) (N = 3; **P < 0.01 and ****P < 0.0001).

The majority of women diagnosed with advanced-stage epithelial OC experience tumor recurrence associated with chemoresistance (29). The above results indicated a role for both ILK and Fzd7 in platinum resistance. Tumor specimens from patients diagnosed with advanced (grade 3, stage 3) serous OC were divided in responders and non-responders to platinum–taxane therapy and analyzed in the ROC plotter database (30). Mann–Whitney test and ROC analysis demonstrated that higher than median expression of *ILK*, *Fzd7*, and/or combination of *ILK-Fzd7* was associated with poor response to platinum–taxane therapy (Fig. 5A–C). KM analysis showed that higher than median expression levels for *ILK, Fzd7*, and/or combination of *ILK-Fzd7*, correlated with poor PFS in patients diagnosed with advanced (grade 3, stage 3 + 4) serous OC and received platinum–taxane therapy (Fig. 5D–F), further supporting a prognostic/predictive role of ILK and Fzd7 in HGSOC patients in response to chemotherapy. To investigate the underlying mechanism of the Fzd7/ILK axis in chemoresistance, cell lines derived from a patient diagnosed with HGSOC (PEA1, platinum sensitive (S)) and from the same patient with acquired platinum resistance (PEA2, platinum resistant (R)) were utilized. In addition, OC-R cell lines (OVCAR-3-R and HEY-A8-R) were generated by continuous *in vitro* exposure to platinum at IC_50_ concentrations (IC_50_ = 4.2 μM for OVCAR-3 and IC50 = 6.1 μM HEY-A8 cells) (Fig. S5A). Increased expression of p-ILK^Ser246^ and the active ILK-target p-AKT^Ser473^ were observed in PEA2, OVCAR-3-R and HEY-A8-R cells compared to the S cell counterparts (Fig. 5G, Fig. S5A). Furthermore, Fzd7 expression and A-β-catenin levels were increased in the R compared to the S cells (Fig. 5H, Fig. S5B). In addition, ILK inhibition by cpd-22 or Fzd7-KD blocked spheroid proliferation and colony formation (Fig. 5I,J) and increased the sensitivity to platinum compared to controls (Fig. 5K,L).

**Figure 6:**
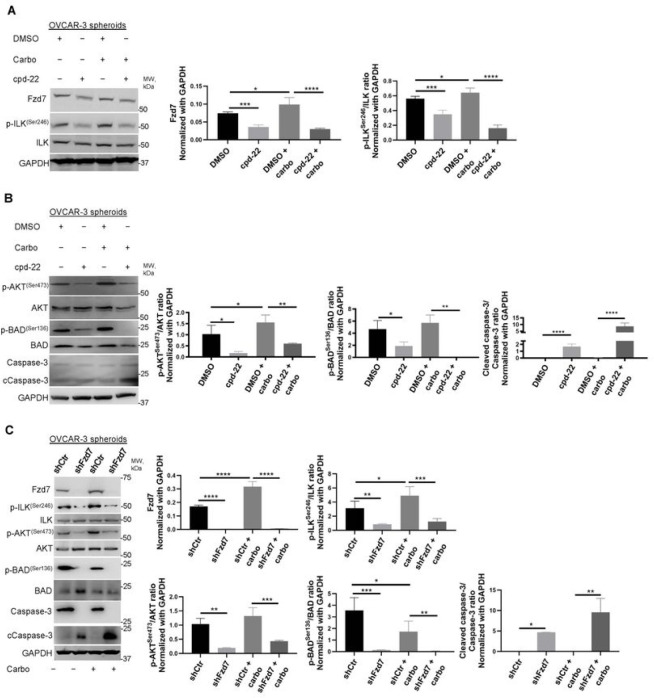
Fzd7-KD and ILK inhibition activate pro-apoptotic signaling in OC spheroids treated with carboplatin. **A, B** WB for Fzd7, p-ILK^Ser246^, ILK, p-AKT^Ser473^, AKT, p-Bad^Ser136^, Bad, Ccaspase-3, caspase-3, and GAPDH in HGSOC OVCAR-3 cells grown as spheroids and treated with DMSO control or cpd-22 in combination or not with carboplatin (double IC_50_) (left panel). Densitometry quantifies Fzd7 expression levels and p-ILK^Ser246^/ILK, p-AKT^Ser473^/AKT, p-Bad^Ser136^/Bad, and Ccaspase-3/caspase-3 ratio (right panel) (N = 3; *P < 0.05, **P < 0.01, ***P < 0.001, and ****P < 0.0001). **C**. WB for Fzd7, p-ILK^Ser246^, ILK, p-AKT^Ser473^, AKT, p-Bad^Ser136^, Bad, Ccaspase-3, caspase-3, and GAPDH in HGSOC shCtr or shFzd7 (clone #2) OVCAR-3 cells grown as spheroids and treated with or not carboplatin (double IC_50_) (left panel). Densitometry quantifies Fzd7 expression levels and p-ILK^Ser246^/ILK, p-AKT^Ser473^/AKT, p-Bad^Ser136^/Bad, and Ccaspase-3/caspase-3 ratio (right panel) (N = 3; *P < 0.05, **P < 0.01, ***P < 0.001, and ****P < 0.0001).

To investigate the mechanism underlying Fzd7/ILK axis in chemoresistance, anti-apoptotic signaling through AKT, one of the known ILK targets (21), was examined. Carboplatin treatment increased p-ILK^Ser246^, p-AKT^Ser473^, and Fzd7 levels in OC spheroids compared to controls (Fig. 6A–C), which were inhibited by cpd-22 treatment or Fzd-7-KD (Fig. 6A–C). Activation of p-AKT^Ser473^ inhibits the pro-apoptotic activity of Bad by promoting phosphorylation at Ser136 (31). Cpd-22 and Fzd7-KD decreased p-BAD^Ser136^ levels in platinum treated OC spheroids and increased cleaved caspase 3 (Fig. 6B,C), enhancing the pro-apoptotic signaling prompted by platinum treatment.

### ILK inhibition targets the platinum resistant OCSC population and improves response to chemotherapy.

**Figure 7:**
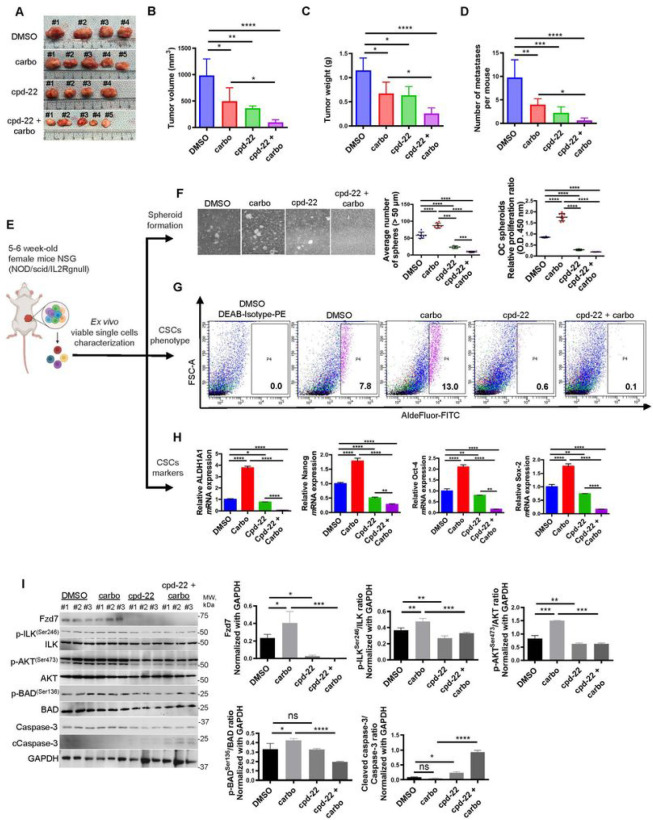
ILK functional inhibition in combination with carboplatin effectively reduces the platinum resistant OCSC phenotype. **A-D.** Tumor morphology, volumes, weights, and numbers of peritoneal metastases derived from HEY-A8 cells injected intraperitoneally in NSG mice treated with DMSO, cpd-22, carboplatin and/or combination (n=5 per group). Data are shown as means ± s.e.m. Significant differences are marked (N = 5 per group; *P < 0.05, **P < 0.01, ***P < 0.001, and **** P< 0.0001). **E**. Graphical representation of ex vivo single cell characterization. **F**. Spheroid morphology (upper panel), CCK-8 proliferation assay (lower left panel) and count (lower right panel) of OC cells isolated from xenografts and grown ex vivo (N = 3; ***P < 0.001 and ****P < 0.0001). **G**. Percentage of ALDH^+^ cells in DMSO control, DMSO + carboplatin-, cpd-22−, cdp-22 + carboplatin-treated xenografts. Cells were isolated by mechanical and enzymatic digestion and ALDH^+^ cells were detected by FACS. **H**. *ALDH1A1*, *Nanog, Sox-2,* and *Oct-4 m*RNA levels measured by qRT-PCR in OC cells isolated from tumors treated with vehicle, cpd-22 alone or in combination with carboplatin (N = 3; *P < 0.05, **P < 0.01, and ****P < 0.0001). **I**. WB for p-FAK^Tyr576/577^, FAK, Fzd7, p-ILK^Ser246^, ILK, p-AKT^Ser473^, AKT, p-BAD^Ser136^, Bad, Ccaspase-3, caspase-3, and GAPDH in whole protein extract from single cells isolated from tumors and grown as spheroids (left panel) (N = 3 per animal group). Densitometry quantifies Fzd7 expression levels and p-FAK^Tyr576/577^/FAK, p-ILK^Ser246^/ILK, p-AKT^Ser473^/AKT, p-Bad^Ser136^/Bad, and Ccaspase-3/caspase-3 ratio (right panel) (N = 3; *P < 0.05, **P < 0.01, ***P < 0.001, and ****P < 0.0001).

In OC, spheroids contribute to ip dissemination and metastasis (5, 32). We examined the effects of cpd-22 on tumor formation and dissemination alone or in combination with platinum in an ip OC xenograft model. Tumor volume, weight, and the number of peritoneal implants were significantly decreased in xenografts treated with either drug alone compared to control cells (Fig. 7A–D). However, the combination of cpd-22 plus carboplatin was significantly more effective in decreasing tumor volume and weight and inhibiting the number of implants compared to carboplatin alone (Fig. 7A–D). Cpd-22 was well tolerated, indicated by no overt signs of toxicity or loss of final body weight at time of sacrifice compared to initial body weight (Fig. S6), in agreement with a previous study (27).

Next, tumors from mice treated with either vehicle, carboplatin, or cpd-22 alone or combination of cpd-22 with carboplatin were dissociated to single-cell suspension at the end of treatment and cells were analyzed for Aldefluor positivity, CSC markers, survival signaling pathways and spheroid formation ability (Fig. 7E). Compared to vehicle-treated mice, single cells isolated from carboplatin treated mice showed increased spheroid formation (Fig. 7F) and expression of stemness markers (*ALDH1A1, Nanog, Oct-4, Sox-2*) (Fig. 7G,H). Interestingly, decreased spheroid proliferation and CSC signature genes were observed in cells isolated from mice treated with cpd-22 alone compared to vehicle or carboplatin alone (Fig. 7F–H). Spheroid formation ability (Fig. 7F) and expression of the CSC markers (Fig. 7G, H) were further decreased by the combination of cpd-22 plus carboplatin compared to vehicle alone.

To examine the functional consequence of blocking outside in signaling by ILK on the OCSC population, cells grown as spheroids from carboplatin treated tumors were examined. Increased levels of Fzd7, active p-ILK^Ser246^, and active p-AKT^Ser473^ were observed compared to vehicle (Fig. 7I). Both cpd-22 alone and moreover the combination of cpd-22 plus carboplatin decreased p-ILK^Ser246^, p-AKT^Ser473^, Fzd7 and the activation of pro-apoptotic pathways, demonstrated by decreased phosphorylation of Bad at Ser136, loss of Bad inhibition as determined by increased cleaved caspase-3 levels (Fig. 7I). Collectively, our data support the targeting of ILK in combination with chemotherapy as promising intervention to decrease the chemoresistant OCSC population and improve response to platinum.

## Discussion

**Figure 8:**
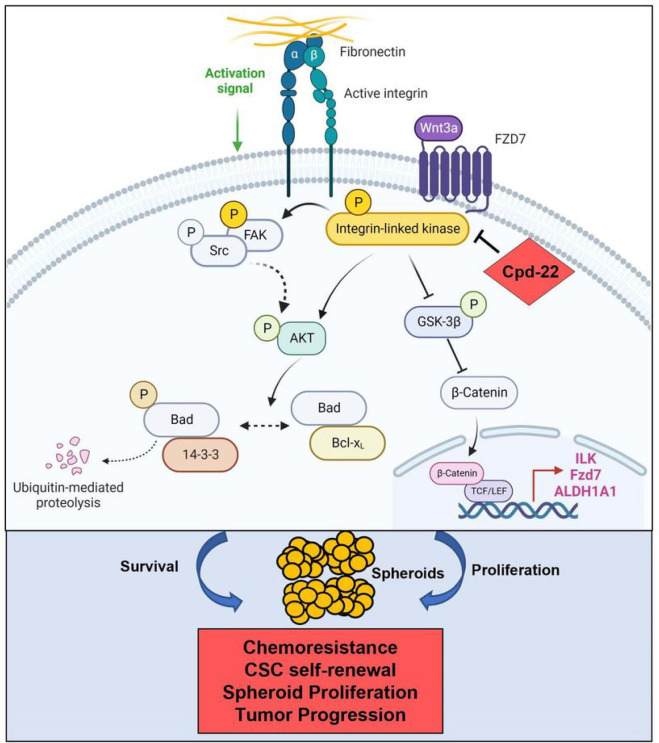
Graphical representation of ILK-Fzd7-mediated OCSC survival and spheroid formation. Fzd7 receives inputs from the ECM and in turn translates a complex “outside in” signaling through ILK/AKT axis that both amplifies the proliferative Wnt/β-catenin pathway and regulates cell survival. ILK inhibition through cpd-22 disrupts both the proliferative and pro-survivals inputs, thus sensitizing OCSCs to platinum and blocking OC progression.

Spheroids formation is accompanied by distinct changes at the interface between tumor cells and ECM allowing OC cells to attach, invade, and grow (33). The ability of cancer cells to survive as spheroids has been linked to a side subpopulation of OCSCs that are associated with chemo-resistance and tumor relapse (5, 34–36). Previously, we demonstrated that the crosstalk between FN and integrin β1 was implicated in OC adhesion to the ECM and outside in signaling through ILK (12). Here, we report a mechanism by which ILK activation drives the OCSC phenotype and disease progression. We show that interaction of active ILK with FN, integrins and Fzd7 plays a crucial role in supporting the survival of OCSCs by promoting spheroid formation and resistance to platinum through the activation of both anti-apoptotic programs and the oncogenic β-catenin signaling (Fig. 8).

Several elements of novelty can be found in our study. First, we showed that OC harbors active p-ILK^Ser246^, consistent with aberrant FN secretion and integrin β1 expression (12), ILK expression is specific to ALDH^+^/CD133^+^ stem cells and up-regulation of the CSC marker ALDH1A1 correlates with ILK and poor patient outcomes. The data agree with previous results linking ECM-re-arrangement and FA complexes formation with the ALDH^+^ stem-like phenotype and tumor progression (37). Considering that CSCs are largely responsible for the considerable complexity and organ specificity of metastasis (38), the findings provide new insight on the impact of ILK expression in the pro-metastatic OCSCs behavior, as strong active p-ILK^Ser246^ in metastatic samples was linked to poor PFS and OS. In addition, our in vivo studies demonstrate for the first time that ILK inhibition by cpd-22 inhibited stemness phenotypes. Taken together with data showing the strong ILK correlation with the CSC marker ALDH1A1, this study mechanistically links the formation of a specialized ECM microenvironment with the pro-metastatic behavior of the stem-like phenotype.

Second, our data demonstrated a direct link between Fzd7 and ILK activation. Fzd7 has been proposed as a novel stem cell-specific receptor with an important role in embryonic stem cell self-renewal capacity (39, 40) and chemo-resistance of gastric cancer side population cells (41). In OC, Fzd7 expression is increased in the chemo-resistant stem-A subtype and correlated with stemness and poor clinical prognosis (12, 24, 42). The canonical Wnt/β-catenin pathway is responsive to matrix assembly (15) and drives CSC maintenance through Fzd7 up-regulation (12), demonstrating a possible link between Fzd7 and matrix re-organization. Fzd7 significantly co-localized with active p-ILK^Ser246^ and that the presence of Wnt-3A was critical in both the increased expression of Fzd7 and ILK and the formation of active Fzd7/p-ILK^Ser246^ clusters in primary OC cells. Further, abnormal ILK activation strongly correlated with Fzd7 expression and was associated with distant organ metastasis, advanced clinical stages, and poor clinical prognosis, supporting the functional relevance of Fzd7/ILK axis in mediating outside in transduction signals and OC progression.

Third, to better understand the link between Wnt-3A treatment and Fzd7/ILK up-regulation, we investigated whether *ILK* is a possible target of the β-catenin transcriptional complex. It has been reported that the nuclear accumulation of β-catenin leads to an up-regulation of Wnt and ECM target genes, such as *Fzd7* (28) and *FN1* (43). We also reported transcriptional regulation of the OCSC marker *ALDH1A1* by β-catenin (6). Other studies showed the canonical Wnt signaling strictly regulated by ILK activation and frizzled-1 expression (44). The findings indicate that the formation of active Fzd7/pILK^Ser246^ clusters in OC spheroids leads to nuclear β-catenin translocation with transcriptional up-regulation of *Fzd7, ILK*, and *ALDH1A1* target genes, thus providing a “feed-forward” signaling that supports OCSC properties.

Fourth, we demonstrated that Fzd7/ILK axis leads to β-catenin activation in chemoresistant HGSOC cells and tissues and promotes pro-survival signaling through escape from apoptosis by activating p-AKT^Ser473^. Previous studies have associated cell adhesion, ECM secretion and deposition with chemoresistance in spheroids (45–47), attributing this phenomenon to decreased penetrance of drugs within spheroids and the presence of resident CSCs (5, 48–50). However, the molecular mechanisms leading to spheroid proliferation and OCSCs survival remain to be fully elucidated. We report for the first time that platinum resistant HGSOC cells express higher levels of Fzd7, active p-ILK^Ser246^, and active p-AKT^Ser473^ when compared to the platinum sensitive counterparts; furthermore, ILK and Fzd7 inhibition increase sensitivity to platinum by specifically targeting the chemoresistant CSC population by blocking spheroid formation and capacity to form colonies. Pro-survival factors, such as AKT, inhibit the pro-apoptotic activity of Bad by promoting its phosphorylation at Ser136 and consequent degradation by 14-3-3 proteins (51). Inhibition of both ILK ad Fzd7 reduced the levels of p-Bax^Ser136^ correlating with Bax activation and increased levels of cleaves caspase-3, resulting in downstream events involved in cell death.

Lastly, the complexity of players and signaling pathways involved regulate not only cellular survival processes, but also CSC proliferation, indicating the disruption of Fzd7/ILK axis as a possible therapeutic target, in agreement with a previous study showing that cpd-22 reduced invasiveness of OC cells (52) and that ILK-KD blocked OC cells growth by inducing cell cycle arrest and apoptosis, decreased OC cells invasion and migration, and delayed tumor formation in OC mouse model (22, 53–55).

## Conclusions

Our preclinical study support development of new strategies aimed at targeting the Fzd7/ILK axis in combination with current chemotherapeutics. Disrupting ILK-Fzd7 clusters may represent a potential therapeutic strategy to eradicate OCSCs and improve treatment outcomes for OC patients.

## Data Availability

Publicly available data were retrieved from the TCGA OC database (Ovarian Serous Cystadenocarcinoma (TCGA, Nature 2011) and the clinical information associated with these samples were obtained from cBioPortal (http://www.cbioportal.org). Survival analysis was performed in advanced HGSOC patients (stage: 3 + 4, grade: 3) using publicly available datasets through Kaplan-Meier plotter and OvMark system. Gene expression and response was linked to therapy by using publicly available transcriptome-level data of ovarian responders and non-responders to platinum–taxane therapy and analyzed in the ROC plotter database. All data generated or analyzed during this study are included within the article and its Supporting Information.

## List of Abbreviations

α: alpha
Akt: Protein kinase B
ALDH1A1: Aldehyde dehydrogenase 1 family member A1
Β: Beta
Bad: BCL2 associated agonist of cell death
CSC: Cancer Stem Cells
Kit: The c-kit protooncogene
CD133: Prominin-1
Co-IP: Co-immunoprecipitation
Cpd-22: Compound 22
DEAB: N,N-diethylaminobenzaldehyde
DMSO: Dimethyl sulfoxide
ECM: Extracellular Matrix
FA: Focal adhesion
FAK: Focal adhesion kinase
FACS: Fluorescence-activated cell sorting
FN: Fibronectin
FZD: Frizzled Receptor
GAPDH: Glyceraldehyde-3-phosphate dehydrogenase
GSK-3α/β: Glycogen synthase kinase-3*α*/*β*
HGSOC: High Grade Serous Ovarian Cancer
IF: Immunofluorescence
IHC: Immunohistochemistry
ILK: Integrin-linked kinase
IP: Intraperitoneal
KD: Knock down
mRNA: Messenger Ribonucleic Acid
Myc: V-myc myelocytomatosis viral oncogene homolog (avian)
Nanog: Homeobox transcription factor Nanog
NSG: Non-obese diabetic/Severe combined immunodeficiency/IL2rγnull
Oct-4: Octamer-binding transcription factor 4
OC: Ovarian Cancer
OS: Overall survival
PCR: Polymerase Chain Reaction
PFS: Progression-free survival
RT: Realtime
Sc: Subcutaneous
Sox-2: SRY-Box Transcription Factor 2
TCF/LEF: T-cell factor/lymphoid enhancer factor
TCGA: The Tumor Cancer Genome Atlas
TME: Tumor Microenvironment
WB: Western Blot
Wnt: Wingless-type MMTV integration site family
